# Elucidating gene signatures that control the circadian rhythm in cyanobacteria using bioinformatics methods

**DOI:** 10.1186/1471-2105-13-S18-A9

**Published:** 2012-12-14

**Authors:** Tulip Nandu, Meeta Pradhan, Mathew J Palakal

**Affiliations:** 1 School of Informatics, Indiana University-Purdue University, Indianapolis, Indiana, 46204, USA

## Background

The circadian rhythm, or biological “clock,” allows the organism to anticipate and prepare for the changes in the physical environment. Studies have found that the internal clock consists of an array of genes and the protein products they encode, which regulate various physiological processes throughout the body. *Cyanothece sp*. ATCC 51142 is an organism that has both photosynthetic (producing oxygen) and nitrogen fixing ability. It has developed a temporal regulation in which N2 fixation and photosynthesis occur at different times throughout a diurnal cycle with very high levels of CO2 fixation during the light and high levels of N2 fixation in the dark. The mechanisms underlying the circadian rhythm and the signature genes elucidating this mechanism are addressed in this research.

## Objective

The objective is to integrate gene expression data with data and knowledge from prior studies using bibliomics techniques, in the de novo construction of quasi-complete regulatory networks to identify gene signatures in functional motifs and elucidate their role in circadian rhythms in *Cyanothece sp*. ATCC 51142.

## Results

**Figure 1:**
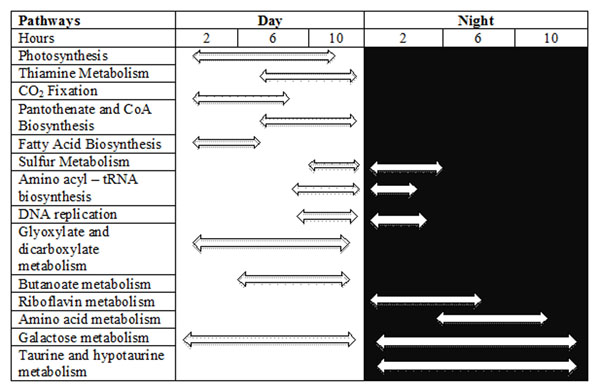
Expression of pathways during the time period

Tables 1 and 2 show the signature genes identified from topological analysis that lead to a specific pathway in *Cyanothece sp*. ATCC 51142. Figure 1 shows the pathways and their peak expression during the time of the day or night depending on the signature genes.

**Table 1:** Signature genes expressed during the day.

Function	Genes
Photosynthesis Cluster	
Photosystem II	psbD2, psbO*, psbA1, psbA3, psbF, psbE, psbY, psbA4*, psbA1*, psbA2*, petA*,
Cytochrome Family	psbV, petB*, petJ
Ferredoxin Type	petF1, petF5,
Carbon Fixation	glcD, glcE, glcF, rbcL
Thiamine Biosynthesis	thiC, thiE, thiL, thiOG
Pantothenate Biosynthesis	panB, panD
CoA biosynthesis	ilvB
Fatty Acid Biosynthesis	accD, fabl, fabG
Amino acyl – tRNA biosynthesis	cysS1*, cysS2*, serS*, pheS*, pheT*, thrS1*, thrS2*, proS*
DNA replication	ligA*, polA*, rnhA*
Glyoxylate and dicarboxylate metabolism	purU, glcD, folD, glcE, glcF
Butanoate Metabolism	pdhA, ilvN, ilvB, gabD

* - Genes that are differentially expressed

**Table 2:** Signature genes expressed during the night

Function	Genes
Sulfur Metabolism	cysC
Amino acid Biosynthesis	cysK, ilvG
Galactose Metabolism	galE1, galE2
Riboflavin Metabolism	ribA, ribC, ribD

## Conclusions and potential implications

The analyses show that most of the top ranked genes in the topological analysis was obtained from text mining. This shows that expression data alone is not a good measure to study the biochemical pathways and signature genes in an organism (specially less studied species).

The algorithms and methodology developed can be extrapolated to any organism, which is less studied to study their gene regulatory elements and also elucidate gene signatures that lead to specific biochemical pathways in a particular organism.

